# ADHERENCE TO THE MEDITERRANEAN DIET IN ELEMENTARY SCHOOL CHILDREN (1^ST^ CYCLE)

**DOI:** 10.1590/1984-0462/2021/39/2019259

**Published:** 2020-08-05

**Authors:** Goreti Filipa Santos Marques, Sara Maria Oliveira Pinto, Ana Catarina Rodrigues da Silva Reis, Tânia Daniela Barbosa Martins, Ana Paula da Conceição, Ana Rita Vieira Pinheiro

**Affiliations:** a Escola Superior de Saúde de Santa Maria, Porto, Portugal.; b Escola de Saúde, Universidade de Aveiro, Portugal.

**Keywords:** Obesity, Children, Diet, Obesidade, Crianças, Padrão alimentar

## Abstract

**Objective:**

To characterize the adherence to the Mediterranean diet (MD) in students from elementary schools in Porto and Maia and analyze its association with sleep hygiene, physical activity, cardiometabolic risk, and school performance.

**Methods:**

This is a cross-sectional study with 891 Portuguese students: 455 boys (51%) and 436 girls (49%), aged between 9 and 11 years old (mean [M]=9.2, standard deviation [SD]=0.4), with an average weight of 35.9 kg (SD=8.1), average height of 1.4 m (SD=0.1), average body mass index (standardized BMI Z score for the pediatric age group) of 0.76 (SD=1.21); 59.5% of them had normal weight and 15.9% were obese. The students filled a questionnaire on the adherence to the Mediterranean Diet (Mediterranean Diet Quality Index in Children and Adolescents [KIDMED]), participated in a socio-demographic interview, and had their anthropometric data collected after their parents signed the informed consent form.

**Results:**

The results suggest high levels of adherence to the MD (77.6%) both in males and females. Using Pearson’s correlation coefficient, we found that the Z score was positively associated to cardiometabolic risk and the starting age of an extracurricular physical activity, and negatively associated to the average hours of sleep on a typical day both in males and females. We also identified a negative relation between KIDMED and the starting age of physical activity.

**Conclusions:**

This study has contributed to the knowledge of adherence to the MD among Portuguese elementary students and correlations with variables associated to a healthier lifestyle (MD, hours of sleep, and physical activity). Future studies should focus their attention on other countries and more heterogeneous samples.

## RESUMO

**Objetivo:**

Caracterizar a adesão à dieta mediterrânica de alunos de escolas primárias do Porto e Maia e analisar a sua associação com a higiene do sono, a atividade física, o risco cardiometabólico e o desempenho escolar.

**Métodos:**

Estudo transversal com 891 alunos portugueses: 455 meninos (51%) e 436 meninas (49%), entre 9 e 11 anos (média [M]= 9,2; desvio padrão [DP]= 0,4), peso médio 35,9 kg (DP= 8,1), estatura média 1,4 cm (DP= 0,1), índice de massa corporal médio (IMC padronizado por escore Z para a faixa etária pediátrica) 0,76 (DP= 1,2), 59,5% de pessoas com peso adequado e 15,9% de obesos. Os alunos completaram o Índice de Qualidade de Adesão à Dieta Mediterrânica em Crianças e Adolescentes (*Mediterranean Diet Quality Index in Children and Adolescents* [KIDMED]) e uma entrevista sociodemográfica e dados antropométricos, após obtenção do consentimento informado dos pais.

**Resultados:**

Os resultados sugerem altos níveis de adesão ao padrão alimentar mediterrânico (77,6%), tanto nos meninos como nas meninas. Recorrendo ao coeficiente de correlação de Pearson, encontramos associação positiva entre o escore Z e o risco cardiometabólico, associação negativa entre o escore Z e a média de horas de sono em um dia típico nos meninos e nas meninas, associação positiva entre o escore Z e a idade de início de uma atividade física extracurricular e associação negativa entre o KIDMED e a idade de início da atividade física.

**Conclusões:**

Este estudo contribuiu para o conhecimento da adesão ao padrão alimentar mediterrânico de alunos do 1º ciclo de escolaridade do Porto e da Maia e correlações com variáveis associadas a um estilo de vida mais saudável (adesão à dieta mediterrânica, horas de sono e atividade física). Estudos futuros deverão focar a sua atenção em outros países e com amostras mais heterogêneas.

## INTRODUCTION

The Mediterranean diet (MD) is considered one of the healthiest dietary patterns in the world, characterized by a nutritional regime consisting of abundant pieces of food of vegetable origin such as bread, pasta, vegetables, fresh fruit, and oilseeds; the use of olive oil as the main source of fat; the moderate consumption of fish, poultry, dairy products, and eggs; the intake of small amounts of red meat; and moderate ingestion of wine, usually during main meals. These are the principles of a healthy diet^1^ that have been associated with better health status, from a cardiovascular, metabolic, and mental point of view.^2^

Adherence to the MD in children and adolescents still lacks investigation, but some studies suggest that this dietary pattern is a protective factor for overweight and obesity, contributing to reduce the risks of disease and morbidity associated with a poor^3^ diet, and preventing the onset of respiratory diseases such as asthma and allergies.^4^

In this sense, it is important to alert to current problems related to childhood obesity, which is a major public health issue.^5^ Estimates indicate that 200 million children are overweight, and 40 to 50 million are obese worldwide. In Portugal, this problem affects all age groups and reaches alarming numbers.^6^

According to the 2016 and 2017 studies of the Portuguese Association Against Childhood Obesity, approximately one out of three children are overweight; however, this condition is not usually restricted to childhood, in other words, overweight or obese children will become overweight or obese adolescents and/or adults, anticipating some complications previously only found in adulthood, such as diabetes, hypertension, cardiovascular disease, and even oncological diseases, among others.^7^ These indicators demonstrate the need to implement targeted interventions not only for children but also for parents, educators/teachers, and the community in general. Interestingly, we have seen a steady increase, at early ages, of non-communicable diseases, often silent, associated with lifestyle-related modifiable risk factors. In this scenario, it is necessary to understand the characteristics of the life habits of children and promote the positive ones to improve the health of the pediatric population.

To this end, a research group was created under the project **For More Health** developed by the Santa Maria Health School (Escola Superior de Saúde de Santa Maria - ESSSM), in partnership with the School of Nutrition and Food Sciences of the University of Porto (Faculdade de Ciências da Nutrição e Alimentação da Universidade do Porto - FCNAUP), the Regional Health Administration of the North (Administração Regional de Saúde do Norte - ARS Norte), the Health Center Network (Agrupamento de Centros de Saúde - ACES) from Maia/Valongo, the School of Nursing São José de Cluny, and the School of Nursing Doutor José Timothy Montalvão Machado (project funded by the NORTH-01-0145-FEDER-024116).

The For More Health project aims to characterize the lifestyles of school children and estimate the prevalence of overweight and obesity.

The objectives of this study are to investigate the level of adherence to MD in a sample of 891 Portuguese students from the 1^st^ cycle of public and private elementary schools in the municipalities of Porto and Maia through the questionnaire on the adherence to the MD (Mediterranean Diet Quality Index in Children and Adolescents [KIDMED]) and analyze the associations with variables related to sleep hygiene, the practice of physical activity, cardiometabolic risk, and school performance.

## METHOD

This is a descriptive cross-sectional study based on quantitative analysis techniques with a convenience sample consisting of 891 students enrolled in the 4^th^ grade of the 1^st^ cycle of public and private elementary schools in the municipalities of Maia and Porto (urban environment) in a universe of 1,200 students (about 25% of the parents of eligible students refused to take part in this research). The mean age of the participants in this study was 9.2 years (9 years, standard deviation [SD]=0.4, range 9-11). Table 1 presents the sociodemographic and anthropometric characteristics of the students (Results section).

**Table 1:** Sociodemographic and anthropometric characteristics of the participants.

	Female (n=436)		Male (n=455)		Total (n=891)	
	M	SD	M	SD	M	SD
Age (years)	9.2	0.4	9.2	0.4	9.2	0.4
Weight (kg)	36.2	8.2	35.6	8.0	35.9	8.1
Height (m)	1.4	0.1	1.4	0.1	1.4	0.1
BMI (Z score)	0.7	1.2	0.8	1.2	0.8	1.2
KIDMED score	9.9	1.9	9.4	2.1	9.7	1.9
Cardiometabolic risk	0.5	0.1	0.5	0.1	0.5	0.1
	n	%	n	%	n	%
BMI categorized						
Normal weight	243	62	228	57	471	59.5
Overweight	97	24.7	93	23.3	190	24
Obesity	51	13	75	18.8	126	15.9
KIDMED (adherence levels) (n=891)						
High (≥8)	367	89.5	339	82.3	706	77.6
Moderate (4-7)	43	10.5	70	17	113	13.7
Poor (≤3)	0	0	3	0.7	3	0.4

M: mean; SD: standard deviation; BMI: body mass index; KIDMED: Mediterranean Diet Quality Index in Children and Adolescents.

The instruments used included a sociodemographic and social characterization form and KIDMED.

The sociodemographic and social characterization form (informed consent form signed by parents authorizing the child’s participation) included information about the child’s gender and age, the parents’ gender, age, and profession/occupation, as well as the living environment, household composition, and housing characteristics (e.g., number of rooms and living rooms). The questions covered the characteristics and behaviors of the child related to sleep pattern, the practice of physical activity, and sedentary hours (television, phone, tablet, and computer measured in minutes per day). A part of the questionnaire was reserved for the teacher to provide information about the school performance of each participant in the areas of Portuguese and Mathematics, and an assessment of the children’s knowledge about dietary patterns and physical activity.

We also evaluated and recorded the height (m) of the children using a portable stadiometer (Seca^®^, Tanita Europe BV, Amsterdam, The Netherlands) and assessed their body composition by weighing (kg) them with a bioimpedance scale (Tanita Segmental Body Composition BC-601^®^, Tanita Europe BV, Amsterdam, The Netherlands) and measuring their waist circumference (cm) with a flexible measuring tape (Seca^®^, Tanita Europe BV, Amsterdam, The Netherlands). For the recording of weight, height, and percentage of fat mass, the value was rounded to the nearest 0.1. The techniques adopted for the anthropometric evaluation followed the criteria proposed by the International Society for the Advancement of Kinanthropometry (ISAK). A 24-hour food recall was also administered to determine energy consumption (total energy intake, fat, carbohydrates, protein, saturated fat, and simple sugars).

KIDMED is a questionnaire consisting of 16 questions that aims to analyze the consumption and daily intake frequency of various food items. The instrument was originally developed to assess the level of adherence to the MD in Spanish children and adolescents aged two to 24 years.^3^ The sum of its values ranges from zero to 12 points, allowing us to classify the adherence to the MD in three levels: level one - high adherence (≥8 points), level two - moderate adherence (4-7 points), and level three - poor adherence (≤3 points).^3^ The version used in this study was the one adapted for the Portuguese population by Serra-Majem and collaborators.^3^

Data were collected during January and July 2018 in several public and private elementary schools in the municipalities of Porto and Maia. The study received formal approval from the National Data Protection Commission (NDPC - Ethics Committee no. 1704/2015). The research team presented the study to the various selected schools in December 2018, and parents and teachers were informed about the objectives of the project during the meetings scheduled for that purpose. After obtaining the informed consent form from the parents authorizing the participation of their children, the first step was to collect data from the 891 participants, whose characteristics are presented in Table 1 (Results section). The research team gathered the information in a room intended for this purpose in each school, always ensuring the privacy of each child who participated in the study.

We selected 4^th^-grade children because a pilot study had already been developed with 3^rd^-grade children, and, at this stage of the investigation, the researchers planned to test the data collection instrument and the effectiveness of a targeted intervention.

All participants watched an awareness-raising video on the promotion of healthy lifestyles that addressed the themes of healthy eating, physical activity, and sleep hygiene, before the administration of the questionnaire.

The results for the classification of the children’s nutritional status were expressed as Z scores (standardized BMI for the pediatric age group), calculated using the WHO AnthroPlus^®^ software 15. Subsequently, the participants were grouped into five categories: extreme underweight (Z score less than -3), underweight (Z score between -3 and -2), normal weight (Z score between -2 and +1), overweight (Z score between +1 and +2), and obesity (Z score greater than +2).

The quantitative data analysis was performed in the software *Statistical Package for the Social Sciences* (SPSS), version 25. The descriptive analysis of the study variables included frequencies, means, and standard deviation. In the inferential analysis, researchers used the chi-square test to check the association between the responses given to each item of KIDMED and gender (nominal variables) and Pearson’s correlation coefficient to test the relationships between the quantitative variables.

## RESULTS

A total of 891 students enrolled in the 4^th^ grade of the 1^st^ cycle of public and private elementary schools in the municipalities of Maia and Porto participated in this study; their mean age was 9 years (SD=0.4, range 9-11). The average weight and height were respectively 35.6 kg (SD=8.0) and 1.4 m (SD=0.1) for boys and 36.2 kg (SD=8.2) and 1.4 m (SD=0.1) for girls.

Table 1 describes these characteristics. Regarding BMI (Z score), this indicator was higher than in girls, corresponding to 0.8 (SD=1.2), and the cardiometabolic risk found in both genders was 0.5 (SD=0.1). Among the participants, 59.5% had normal weight, and 15.9% were obese. The adherence to the MD was high in 77.6% of the students.

Table 2 presents the answers obtained in the KIDMED questionnaire according to gender and the total sample.

**Table 2:** Responses obtained in the Questionnaire of Adherence to the Mediterranean Diet according to gender.

KIDMED	Female (n=436)	Male (n=455)	Total (n=891)	chi-square	p-value
	%	%	%		
1. Do you usually (four or more days a week) have breakfast?	97.5	98	97.8	0.23	n.s.
2. Do you have a dairy product (yogurt, milk, etc.) for breakfast?	91.2	89.9	90.5	0.46	n.s.
3. Do you eat pastry/confectionery products for breakfast?	12.4	15.2	12.5	1.33	n.s.
4. Do you eat cereals and their products (bread, breakfast cereals) for breakfast?	86.6	82.8	84.7	2.31	n.s.
5. Do you eat at least one piece of fruit every day?	92	82.6	87.3	16.23	<0.001
6. Do you eat more than one piece of fruit every day?	65.9	54.2	60.1	11.76	<0.001
7. Do you regularly (four or more days a week) eat vegetables, either raw (lettuce, tomato, etc) or cooked (broccoli, cabbage, etc.), once a day?	79.3	76.8	78.1	0.76	n.s.
8. Do you regularly (four or more days a week) eat raw or cooked vegetables more than once a day?	59.1	56.4	57.8	0.62	n.s.
9. Do you eat fish regularly (at least twice a week)?	82	76.1	79	4.34	<0.05
10. Do you like legumes (beans, grains, peas, etc.) and have them more than once a week?	76.4	69.6	73	4.88	<0.05
11. Do you eat pasta or rice almost every day (5 or more days a week)?	84.2	84.1	84.1	0.002	n.s.
12. Do you eat dried fruits (nuts, hazelnuts, etc.) regularly (at least two to three times a week)?	29.0	28.3	28.6	0.05	n.s.
13. Do you use olive oil in your home?	95.1	94.2	94.7	0.35	n.s.
14. Do you eat two yogurts and/or a slice of cheese (40 g) daily?	51.3	51.7	51.5	0.01	n.s.
15. Do you eat candies and snacks several times a day?	9.2	10.1	9.7	0.19	n.s.
16. Do you go to a fast-food restaurant more than once a week?	6.6	10.1	8.4	3.44	n.s.

n.s.: not significant; KIDMED: Mediterranean Diet Quality Index in Children and Adolescents.

Analyzing the responses by gender, we found that they were equivalent between boys and girls. Among the positive results, we underline the habit of having breakfast every day; the use of olive oil; and the consumption of at least one piece of fruit per day, as well as raw or cooked vegetables, dairy products, fish, and cereals and their products. It is important to highlight the lower number of responses given by the participants concerning the intake of a second piece of fruit per day, raw or cooked vegetables more than once a day, and dried fruits.

In addition to the characterization of the responses given in the KIDMED questionnaire, the answers of each item were analyzed according to gender to check for associations between these variables. There was a statistically significant association between gender and the answers given in questions five, nine, and ten.

We found that girls consume fruit at least once a day, have more than one piece of fruit a day, eat fish at least twice a week, and like and consume vegetables more than once a week in greater proportion than boys.

Table 3 presents the Z score associations, hours of sleep (on the night before and on average on a typical day), physical activity in hours per week, sedentary hours (television, phone, tablet, and computer measured in minutes per day), total energy intake (calories consumed in the previous day), cardiometabolic risk, number of daily meals, performance in Portuguese and Mathematics, and student’s concentration in school activities (responses obtained from the teachers responsible for the class) according to the female gender, while Table 4 shows the same information according to the male gender.

**Table 3:** Pearson’s correlation coefficients between the Z score, hours of sleep, physical activity, total energy value, cardiometabolic risk, daily meals, Portuguese and Mathematics performance, and student’s concentration in school activities (females).

Female gender (n=436)														
(1) Z score	-													
(2) How many hours did you sleep yesterday?	n.s.	-												
(3) On average, how many hours do you sleep?	-0.18**	0.59**	-											
(4) How often do you practice physical activity?	n.s.	n.s.	n.s.	-										
(5) Starting age of an extracurricular activity	n.s.	n.s.	n.s.	n.s.	-									
(6) Total hours of physical activity	n.s.	n.s.	n.s.	0.14*	-0.15*	-								
(7) Total sedentary hours	n.s.	n.s.	-0.14*	n.s.	n.s.	n.s.	-							
(8) Total energy value	n.s.	n.s.	n.s.	n.s.	n.s.	n.s.	n.s.	-						
(9) Cardiometabolic risk	0.69**	n.s.	n.s.	n.s.	n.s.	n.s.	n.s.	n.s.	-					
(10) KIDMED total score	n.s.	n.s.	n.s.	n.s.	n.s.	0.15**	-0.15**	n.s.	n.s.	-				
(11) Number of daily meals	n.s.	n.s.	n.s.	n.s.	n.s.	n.s.	0.10*	0.30**	n.s.	n.s.	-			
(12) Academic achievement in Portuguese	n.s.	-0.12*	n.s.	n.s.	-0.13*	0.12*	n.s.	n.s.	-0.18**	0.13*	n.s.	-		
(13) Academic achievement in Mathematics	n.s.	-0.17**	n.s.	n.s.	n.s.	0.13*	n.s.	n.s.	-0.19**	0.13*	n.s.	0.82**	-	
(14) Concentration in class	n.s.	-0.12*	n.s.	n.s.	n.s.	n.s.	n.s.	n.s.	n.s.	0.11*	n.s.	0.59**	0.58**	-
	(1)	(2)	(3)	(4)	(5)	(6)	(7)	(8)	(9)	(10)	(11)	(12)	(13)	(14)

*p<0.05; **p<0.01; n.s.: not significant; KIDMED: Mediterranean Diet Quality Index in Children and Adolescents.

**Table 4:** Pearson’s correlation coefficients between the Z score, hours of sleep, physical activity, total energy value, cardiometabolic risk, daily meals, Portuguese and Mathematics performance, and student’s concentration in school activities (males).

Male gender (n=455)														
Z score	-													
How many hours did you sleep yesterday?	-0.10*	-												
On average, how many hours do you sleep?	-0.12*	0.50**	-											
How often do you practice physical activity?	n.s.	n.s.	n.s.	-										
Starting age of an extracurricular activity	0.13*	n.s.	n.s.	n.s.	-									
Total hours of physical activity	n.s.	n.s.	0.20**	0.20**	n.s.	-								
Total sedentary hours	n.s.	-0.16**	n.s.	n.s.	n.s.	n.s.	-							
Total energy value	n.s.	n.s.	n.s.	n.s.	n.s.	n.s.	n.s.	-						
Cardiometabolic risk	0.73**	-0.12*	n.s.	n.s.	0.16**	n.s.	-0.11*	0.11*	-					
KIDMED total score	n.s.	n.s.	n.s.	n.s.	-0.12*	n.s.	n.s.	n.s.	n.s.	-				
Number of daily meals	n.s.	n.s.	n.s.	n.s.	n.s.	n.s.	0.34**	n.s.	0.13*	0.13*	-			
Academic achievement in Portuguese	n.s.	n.s.	n.s.	n.s.	n.s.	n.s.	n.s.	-0.16**	0.17**	n.s.	n.s.	-		
Academic achievement in Mathematics	n.s.	n.s.	n.s.	n.s.	-0.17**	n.s.	0.17**	-0.16**	0.14**	n.s.	n.s.	0.82**	-	
Concentration in class	n.s.	n.s.	n.s.	n.s.	-0.13*	n.s.	n.s.	-0.14**	0.16**	n.s.	n.s.	0.64**	0.63**	-
	(1)	(2)	(3)	(4)	(5)	(6)	(7)	(8)	(9)	(10)	(11)	(12)	(13)	(14)

*p<0.05; **p<0.01; n.s.: not significant; KIDMED: Mediterranean Diet Quality Index in Children and Adolescents.

Table 3 reveals a positive and statistically significant association between hours of sleep in the previous night and the usual hours of sleep and between Z score and cardiometabolic risk, suggesting that higher Z scores increase the probability of cardiometabolic problems in girls, and a negative relationship between the Z score and the average hours of sleep on a typical day. Regarding the hours devoted to physical activity, we identified that this variable is positively associated with the KIDMED total score and negatively with sedentary hours, that is, children who spend more time in sedentary activities (television, phone, tablet, and computer) have lower adherence to MD. With respect to school performance according to the teacher’s opinion, we found an association between achievements in Portuguese and Mathematics, and both have a positive relationship with the student’s concentration in the classroom.

Males (Table 4) showed a statistically significant negative association between the cardiometabolic risk and the hours of sleep in the previous night, that is, the cardiometabolic risk increases when the boys sleep less. They also present a positive and significant association between the hours of sleep in the previous night and the usual hours of sleep, similarly to girls. We identified that the starting age of an extracurricular physical activity had a positive and significant association with the Z score and cardiometabolic risk; these findings suggest that children with higher Z score and cardiometabolic risk started practicing physical activity later. The total energy intake was positively associated with cardiometabolic risk, indicating that greater energy consumption contributes to higher cardiometabolic risk. Z score and cardiometabolic risk presented a positive and significant association, i.e., a higher BMI also contributes to a higher cardiometabolic risk. Concerning the associations found with KIDMED, we detected a negative and significant association with the starting age of the practice of physical activity, that is, the children who most adhere to MD began practicing extracurricular physical activity earlier. In the boys’ group (Table 4), we also found a positive and significant association between the number of daily meals and sedentary hours, suggesting that children who have a diet with more meals during the day spend more hours on the television, phone, tablet, and computer, but adhere more to MD.

We also highlight the negative and significant association of the total energy intake with the school performance in Portuguese, Mathematics, and concentration in classes, indicating that greater energy consumption can lead to lower school performance and lower concentration in academic activities. Finally, we identified the same association pattern in the female gender (Table 3) regarding the correlation between the Portuguese and Mathematics performances and the concentration in classes.

## DISCUSSION

Considering the results gathered from the responses obtained in KIDMED, we found that the dietary pattern in females is very similar to that in males, except for items related to the consumption of fruit once and more than once a day, fish, and vegetables, which are higher in girls.

We identified a high level of adherence (≥8 points) to the MD in males, females, and the total sample (77.6%) when compared to other studies we have mentioned.

Investigations developed in Portugal present high adherence levels - 85% in a sample of students from the 5^th^ grade in Tavira^8^ and 48% in a sample of Northern adolescents.^9^ In a population of adolescents from rural areas (Vila Real), older adolescents and those with healthier lifestyles (more frequent consumers of fruit, less neophobic, no history of smoking, and more physically active) presented greater adherence to MD assessed by KIDMED, thus suggesting the concept of lifestyle clustering according to the authors.^10^ We believe that the differences found in our sample are due to context characteristics. Maia is an urban environment county which is very concerned with the promotion of a healthy lifestyle. Perhaps for this reason, our results are superior to those of other Portuguese studies.

Another relevant study in Portugal is the cohort Epidemiological Health Investigation of Teenagers in Porto (EPiTeen), which began in 2003 with the main objective of understanding how habits and behaviors acquired in adolescence will reflect on their health as adults. This project followed the adolescents who were born in 1990 and attended public and private schools in Porto. Regarding KIDMED in the EPiTeen research, the authors reported a median adherence of five points in 13-year-old adolescents in Porto, similar between boys and girls.

These numbers are lower than the ones found in this study with children of the 4^th^ grade and those of the study with adolescents from a rural environment (median score of three in boys and four in girls).

Other countries, such as Spain (EnKid Study, 1998-2000), showed high levels of adherence to MD (46.4%), but in Greece, these levels were considered low.^3^

We identified several statistically significant associations between the variables Z score, hours of sleep, physical activity, total energy intake, cardiometabolic risk, daily meals, performance in Portuguese and Mathematics, and student’s concentration in school activities, although some have a weak value. In particular, we underline the relationship between the Z score and fewer hours of sleep on a typical day, higher cardiometabolic risk, and late starting age of physical activity in boys and girls. Still, children with greater adherence to MD (KIDMED) tend to practice more hours of physical activity and spend fewer hours on TV, tablet, computer, or phone. In males, we found that the number of meals throughout the day was associated with more sedentary hours and that a higher energy intake contributed to lower school performance and concentration in academic activities. These results point to the importance of adopting healthy lifestyle habits, namely the practice of physical activity, good sleep hygiene, and adequate dietary patterns. Considering the predominant role of sleep in children’s development and growth, with an emphasis on cognitive functions and learning, it is important to promote restorative sleep. Our results suggest a reciprocal relationship between the hours of sleep and the prevalence of obesity, and the increase in BMI was associated with the decrease in hours of sleep (association between the Z score and the average hours of sleep on a typical day), as reported in other studies.^11^ ^,^ ^12^

Research in this area^13^ ^,^ ^14^ has also succeeded in establishing a bidirectional relationship between sleep and physical activity, indicating that the greater the practice of exercises, the greater the duration and quality of sleep. Sleep can potentially contribute to changes in the weight of the pediatric population, since a lower number of hours of rest may result in less availability for physical activity due to fatigue, causing a higher investment in sedentary activities that do not require physical exertion by the children.^15^ The duration and quality of sleep seem to influence the eating habits,^16^ and some studies suggest a relationship between short-term sleep and increased consumption of fats/lipids and snacks outside main meals, leading to a total daily energy intake exceeding the appropriate and desired one. This pattern can persist in adulthood, adding to the risk of obesity-related diseases.^13^

We consider that this study presents strengths associated with the characterization of a sample of 891 4^th^ grade students regarding their eating habits, physical activity, sedentary hours, and sleep patterns. However, we underline that the results should be analyzed taking some limitations into account. These limitations correspond to the collection of data from children, as they may not reflect the full information, and the need to extend the sample. Despite these considerations, we can infer that the students evaluated are an apparently healthy group by the prevalence of normal weight among them.

In short, we concluded that the objectives of this study - to characterize the level of adherence to MD in a sample of 891 Portuguese students from the 1^st^ cycle of public and private elementary schools in the municipalities of Porto and Maia through KIDMED and analyze the associations with variables related to sleep hygiene, the practice of physical activity, cardiometabolic risk, and school performance - were successfully met. Very few studies in Portugal address the intervention in the area of sleep and weight in the pediatric population. Therefore, we alert to the importance of developing nationally representative studies on dietary patterns, sleep hygiene, and physical activity.

In view of the results obtained, we strongly suggest the elaboration of an action plan that promotes and improves the acquisition of healthy lifestyle habits throughout the life cycle and prevents the onset of diseases. It is in this scenario that the project For More Health intends to act, having already developed an application on healthy lifestyle habits and promoted its use in the school environment in a playful way, relying on the collaboration of teachers.
